# Nighttime Primary Headaches in Children: Beyond Hypnic Headache, a Comprehensive Review

**DOI:** 10.3390/life15081198

**Published:** 2025-07-28

**Authors:** Beatrice Baldo, Ilaria Bonemazzi, Antonella Morea, Roberta Rossi, Alessandro Ferretti, Vittorio Sciruicchio, Alessia Raffagnato, Vincenzo Raieli, Antonia Versace, Irene Toldo

**Affiliations:** 1 Juvenile Headache Center, Department of Woman’s and Child’s Health, University of Padua, 35128 Padua, Italy; 2 Children Epilepsy and EEG Center, San Paolo Hospital, ASL Bari, 70132 Bari, Italy; 3 Pediatric Headache Center, Department of Pediatric Emergency, Regina Margherita Children’s Hospital, 10126 Turin, Italy; 4 Pediatrics Unit, Neuroscience, Mental Health and Sense Organs (NESMOS) Department, Faculty of Medicine and Psychology, Sapienza University of Rome, 00198 Rome, Italy; 5 Child and Adolescent Neuropsychiatric Unit, Department of Women’s and Children’s Health, University of Padua, 35128 Padua, Italy; 6 Child Neuropsychiatry Department, Istituto Mediterraneo di Eccellenza Pediatrica-Azienda Ospedaliera di Rilievo Nazionale e di Alta Specializzazione “Civico Di Cristina e Benfratelli” (ISMEP-ARNAS Civico Palermo), 90100 Palermo, Italy

**Keywords:** migraine, cluster headache, hypnic headache, nocturnal awakenings, sleep, children, adolescents

## Abstract

Many headaches at night arise due to primary headache disorders, which occur independently of other symptoms and are not caused by another medical condition. Primary headache disorders with nighttime attacks can include tension-type headaches, migraines, hypnic headaches, and cluster headaches. A hypnic headache is sometimes called an “alarm clock headache” because symptoms tend to arise at the same time of night. Apart from considering primary headaches, secondary causes of nighttime headaches should be considered and ruled out, in particular headaches secondary to intracranial hypertension, temporomandibular joint issues (like bruxism) and sleep apnea. Treatments vary based on headache type but often include a combination of medications and prevention strategies. This review article covers the basics of nighttime primary headaches in children, including pathophysiology, etiology, clinical features of the different forms and their treatment. It will also discuss the differences in headache features between children and adults.

## 1. Introduction

The prevalence of headaches in the pediatric population reaches up to 70%, considerably impacting affected individuals and society. Most pediatric headaches are either primary or secondary to non-serious causes; however, a small percentage are due to potentially life-threatening intracranial conditions [1].

One of the commonly considered red flags in pediatric headaches is a headache that causes nocturnal awakenings, which is frequently regarded as a potential indicator of elevated intracranial pressure, especially when accompanied by additional warning signs such as severe vomiting or neurological abnormalities on neurological examination. This condition may be idiopathic or secondary to another intracranial pathology and can also be life-threatening. Other organic conditions, considered benign, may be associated with headaches and nighttime awakenings, such as bruxism and sleep apnea [2,3]. Nonetheless, it is important to note that up to 25% of primary headaches are associated with night-time awakenings. The relationship between sleep and primary headaches has been extensively studied. Inadequate sleep duration or poor sleep quality are well-recognized triggering factors for migraines. Conversely, a headache itself may lead to varying degrees of sleep disruption and is frequently associated with multiple sleep disturbances in both adults and children [4]. It is thought that sleep disturbances and headaches share a common pathophysiological pathway; however, this relationship is currently poorly understood [5].

This study aims to investigate further primary headaches that are frequently associated with nocturnal awakening, specifically migraines, cluster headaches, and hypnic headaches, including pathophysiology, etiology, clinical features of the different forms, and their treatment. Their diagnostic criteria according to “The International Classification of Headache Disorders 3rd Edition” (ICHD3) are reported below (Table 1) [6].

## 2. Materials and Methods

A systematic search was conducted across PubMed (U.S.A.), Embase (N.L.), MEDLINE (U.S.A.), and the Cochrane Library (U.K.). Search terms included combinations of “pediatric,” “children,” “adolescent,” “migraine,” “headache,” “nocturnal,” “sleep,” “hypnic headache,” and “cluster headache.” The search aimed to identify studies reporting migraine or headache episodes occurring during nighttime sleep in individuals aged 1–18. The inclusion criteria were studies involving participants aged 1–18 years experiencing migraine or headache attacks during nighttime sleep, study designs including randomized controlled trials, cohort studies, case series, case reports, systematic reviews, and meta-analyses, and articles published from 1994 to June 2025 in the English language. The exclusion criteria were studies focusing exclusively on adult populations, non-peer-reviewed articles, editorials, and conference abstracts. For the articles included in the review, the ICHD-3 criteria [6], published in January 2018, were applied in those published after that date; in other cases, previous versions of the ICHD were used, or, in some instances, the criteria were not specified.

## 3. Results

### 3.1. Secondary Headaches

Most pediatric headaches are generally benign and self-limiting conditions that can be classified as either primary or secondary due to non-life-threatening diseases (e.g., upper airway infection, influenza, sinusitis, toxic exposure, mild head trauma, or psychiatric disorders) [7,8].

In a minority of patients (up to 2% of patients who come to the pediatric emergency department due to headache), headaches are secondary to serious life-threatening intracranial disorders (such as central nervous infection, hydrocephalus, intracranial hemorrhage, venous sinus thrombosis, ischemic stroke, brain tumors, and benign intracranial hypertension [9]. The primary causes of emergency department visits for headaches are summarized in Table 2.

The red flags are alerting signs and/or symptoms associated with an increased risk of secondary life-threatening headaches, which should lead to further investigation and force the clinician to perform neuroimaging. The main red flags reported in the literature include neurological examination abnormalities (such as alterations of consciousness, focal deficit, meningism, ataxia, visual deficits, papilledema, cranial nerve palsies, progressive increase of head circumference), seizures, severe vomiting, pain awaking the child from nocturnal sleep, worsening of pain with Valsalva maneuver, sudden onset of headache or worsening/change of a previous headache pattern, lack of familiarity for primary headaches, and age under 3 years. Occipital pain, once considered a risk factor, has not been confirmed as a red flag in later studies [11].

As previously reported, nocturnal awakenings are regarded as red flags in pediatric headaches; nevertheless, their diagnostic accuracy remains a matter of debate according to some studies [12]. Pain that awakens a patient from sleep is traditionally considered a sign of increased intracranial pressure, due to the shift in gravitational forces when moving to a supine position: the ICHD-3 does recommend that if a headache wakes a patient from sleep, other potentially serious causes should be ruled out [3,12]. However, a recent study by Ahmed et al. found that among patients presenting with headache-related sleep interruption or early-morning onset, neuroimaging revealed intracranial abnormalities in only 4% of cases, none of which required urgent intervention or were likely responsible for the nocturnal symptoms [3,11]. These findings suggest that while nocturnal headaches can be alarming, their predictive value for serious conditions is limited in the absence of other warning signs. Nonetheless, previous studies have reported that this symptom occurs in approximately 25% of primary headache cases, and is non-specific, especially if isolated (according to some studies, the more red flags there are, the more serious the underlying cause is) [10,13]. Ultimately, other causes of headache associated with nocturnal wakening need to be excluded, including sleep apnea, nocturnal hypertension, hypoglycemia, and medication overuse headache [12]. A careful differential diagnosis is essential to avoid unnecessary investigations while not overlooking potential secondary causes.

The literature does not report significant differences in the timing of nocturnal onset, duration, or clinical features between primary and secondary headaches with nocturnal onset. The only reported distinctions concern idiopathic intracranial hypertension, which seems to be characterized by a late-night onset [14], and sleep apnea-related headache, which, according to the ICHD-3 criteria, typically presents upon awakening [6].

An additional detail regarding the time of onset is that, in primary headaches, nocturnal awakening due to headaches is typically associated with pain that was already present at the time of falling asleep [11]. These nuances in onset timing may provide helpful, albeit limited, clues in distinguishing between headache types.

National and international guidelines, however, reveal a lack of consensus, suggesting that the clinical implications of headache with nocturnal wakening are not necessarily fully understood. There is a rationale for not routinely performing brain imaging in neurologically intact and otherwise healthy children presenting with a headache upon awakening or a sleep-interrupting headache [12]. A more measured approach, balancing caution with clinical judgment, is thus warranted in these cases.

Given the limited consensus on the accuracy of specific red flags, a thorough patient history becomes essential to guide the clinician’s diagnostic and therapeutic approach. Some of the key aspects to consider in the patient’s history include a family history for primary headaches, a personal history of primary headache or past episodes of acute headache, which might suggest a primary etiology of headache, the presence of an aggravating headache, repeated nocturnal awakenings, neurological diseases, psychiatric disorders, recent story of infection, toxic exposure, that, instead, are indicative of a possible secondary etiology of headache [8,11].

### 3.2. Migraine

Our review confirms the existence of a strong relationship between migraine and sleep in children. This correlation shows bidirectional aspects: some children with migraine experience migraine attacks during nighttime; on the other hand, sleep disorders are reported as the most frequent comorbidity in children with migraine.

#### 3.2.1. Clinical Features of Migraine with Nocturnal Attacks

According to the ICHD-III [6], nocturnal migraines are not a separate diagnostic category but rather a temporal variant of migraine that occurs during sleep, often causing abrupt awakening, and sleep-related symptoms are among the most frequently cited trigger factors. While the exact prevalence of nocturnal migraines in pediatric populations is unknown, studies suggest that up to 20–30% of children with migraine may experience attacks that begin or peak during sleep or early morning hours. Migraine attacks have also been well documented to occur during specific sleep stages. In particular, attacks are more likely to occur during periods of REM sleep and with morning arousals associated with larger amounts of stage III, stage IV, and REM sleep.

Gori et al. [15] conducted a case-control study to estimate sleep quality chronotypes, and the possible circadian timing of attacks in adults and pediatric patients with migraine based on 100 young patients (mean age ± S.D. 38.6 ± 10.4 years; range 23–50 years) suffering from migraine without aura according to the IHS criteria, and 30 healthy controls. Morning and evening type subjects were more represented in migraine patients than in controls and showed a tendency towards worse sleep quality and higher disability. Forty-two percent of patients presented more than 75% of their attacks at night and in the early morning hours, especially from 3 to 7 a.m. [15].

Several hypotheses have been proposed to explain why migraine attacks might preferentially occur at night in some cases:

- Circadian influences: The suprachiasmatic nucleus in the hypothalamus regulates circadian rhythms and interacts with brainstem nuclei involved in pain modulation. Dysregulation may predispose certain individuals to nocturnal attacks. Premonitory symptoms like mood states such as alert, tense, depressed, or tired, and changes in sleep quality have been described to occur up to 2 days before a migraine attack and were hypothesized to be related to a hypothalamic involvement in the prodromic phase of migraine [16,17].
- REM sleep instability: Migraine attacks have been observed to coincide with transitions into or out of REM sleep, a phase during which the brain is highly active and fluctuations in autonomic tone may occur [18].
- Melatonin dysregulation: Reduced nocturnal melatonin levels have been reported in both adults and children with migraine. Melatonin has both antioxidant and anti-nociceptive properties and may act as a migraine preventive agent [19].

#### 3.2.2. Migraine Is Associated with Sleep Disorders

The association between headache and sleep disorders in children and adolescents has been known for several years, and this is particularly evident in migraine. Studies suggest that the link between migraines and sleep disturbances is possibly due to genetic and neurotransmitter imbalances (serotonin and dopamine). These imbalances may cause sleep issues in early life and migraine later [20].

The main studies focusing on the relationship between sleep and migraine are shown in Table 3.

Bruni et al. in 1997 [2] reported a survey that was the first on a wide-based pediatric population, to determine the prevalence of sleep disorders in children with migraines and tension-type headaches, compared to a group of healthy subjects. They included 283 headache patients (164 with migraines and 119 with tension-type headaches) and 893 healthy subjects (Table 3). Data reported a high prevalence of sleep disturbances in headache children with major complaints concerning sleep quality, night awakenings, nocturnal symptoms, and daytime sleepiness; in particular, these problems were prevalent in the migraine group. Migraine children also showed a higher occurrence of sleep breathing problems, sleep talking, bruxism, nightmares, and frightening dreams; these nocturnal symptoms were responsible for a more fragmented sleep in patients with migraines (Table 3).

About 8% of patients with headaches reported nocturnal attacks, and 65% of these were migraine children; patients with nocturnal attacks showed more sleep disorders than patients with diurnal attacks, particularly bedtime struggles, night wakings, parasomnias, and daytime sleepiness [2].

The relationship between bruxism and migraines was analyzed in a case-control study published in 2014 by Masuko et al. [5]. They used polysomnography to investigate the prevalence of bruxism during sleep in children with episodic migraines relative to controls. Twenty-five percent of children with sporadic migraines exhibited bruxism during sleep studies, while no control subjects experienced this phenomenon.

The polysomnographic characteristics in children with migraines were described by Armony Domany et al. in 2019 [21]. They included 256 migraine children referred to a Sleep center over a ten-year period. Their most common presenting sleep symptoms were snoring, sleep onset and sleep maintenance problems, excessive daytime sleepiness, restless sleep, and leg movements. Analysis of polysomnography revealed that 39% had obstructive sleep apnea (OSA), 12.9% had moderate to severe OSA, 16.8% showed periodic leg movements, and 2.7% hypoventilation [20].

In 2008, Bruni [4] investigated the relationships between headache and sleep quality in a large non-clinical population of children and adolescents and evaluated the link between headache and circadian typologies. They recruited 1073 children and adolescents from four schools and classified them into three groups: the migraine group (MG), the non-migraine group (NMG), and the headache-free group (HFG).

Regarding the characteristics of headache, the timing of the attacks in both headache groups was preferentially in the evening (32.86% MG, 40.00% NMG), less frequently in the afternoon (15.71% MG, 18.52% NMG), and during the night (12.86% MG, 11.11% NMG). A clear prevalence of the occurrence of attacks during the morning (upon awakening) was found in the migraine group. MG (12.86%) vs. NMG (4.44%) [v2 = 4.9; *p* < 0.05]. Despite that, regarding the predisposing/causative factors, ‘‘a bad sleep’’ is the primary cause of headache attack reported by both groups (32.86% MG, 31.85% NMG), followed by emotional distress (25.71% MG, 28.89% NMG). Both headache groups revealed significantly more sleep-related problems in comparison to the headache-free group. Furthermore, migraine children showed a higher frequency of sleepiness compared to the other groups and a more pronounced eveningness; the latter finding is very interesting because it has never been reported in children [4].

Another recent study by Voci et al. [22] analyzed the relationship between headache features and sleep in pediatric migraines. They reported data about 140 children with migraines, and more than 70% of these received a diagnosis of a sleep disorder. Patients with sleep disorders showed a higher headache frequency and higher prevalence of vertigo and episodic syndromes that may be associated with migraine. Furthermore, decreased total sleep time, as well as longer sleep onset delay and shorter sleep duration, were associated with a higher frequency of migraine episodes. Lastly, they observed that children with a worse sleep quality were more prone to developing severe migraine attacks and to having lower efficacy in the use of abortive treatment.

The authors revealed that only 5% of studied patients had already received a diagnosis of sleep disorders, and none of them had undergone specific evaluation. According to this observation, they highlighted the importance of performing a careful analysis of sleep habits during the clinical evaluation of migraine patients in order to identify the presence of possible sleep problems [22].

A group [23] conducted a case-control study to evaluate the existence and types of sleep and psychiatric abnormalities in school-age children with migraines. They observed a high incidence of psychiatric and sleep abnormalities in migraine children. The most common psychiatric disorders were anxiety, depressive symptoms, withdrawal, depressed symptoms, social problems, somatic complaints, and attention problems. At the same time, children with migraines experienced decreased sleep quality, excessive daytime sleepiness, and polysomnography abnormalities in the form of decreased total sleep time and sleep efficiency, in addition to increased sleep latency, wake after sleep onset, and a deformed sleep architecture. Furthermore, in these patients, the rate of sleep abnormalities was proportional to the degree of disability caused by the recurrent headache attacks.

#### 3.2.3. Management and Treatment

Studies reported above prove that investigation and treatment of comorbid sleep disorders and sleep hygiene interventions should always be considered in the management of migraine patients, since an improvement in sleep is expected to determine a reduction of headache severity and disability.

In some cases, children may not verbalize pain clearly, especially at younger ages, and may instead be irritable, cry inconsolably, or seek parental comfort during the night; for these reasons, some manifestations related to sleep can be missed or misdiagnosed in migraine children.

Children with migraine, therefore, need a careful assessment of their sleep habits and of any associated sleep disorders, eventually performing a polysomnography if necessary.

As already mentioned, people affected by migraines with nocturnal attacks and/or sleep disorders are prone to experience more frequent and more severe headaches. For these reasons, they are at risk of analgesic abuse, and clinicians should prefer non-pharmacological treatment when managing these patients.

Interventions targeting sleep hygiene have been shown to significantly reduce the frequency and duration of migraine episodes: the literature highlights the importance of promoting healthy sleep practices, such as maintaining regular sleep schedules, establishing consistent bedtime routines, and minimizing screen time before sleep [24]. These lifestyle modifications should be encouraged by clinicians and actively supported by parents and caregivers. In addition to behavioral strategies, cognitive-behavioral therapy focused on sleep and stress management may represent a valuable therapeutic option.

A previous review published on headache analyzed the knowledge about the use of melatonin in the treatment of primary headache disorders [19]. Melatonin has important biological roles, including antioxidant, anti-inflammatory, and chronobiotic effects. It supports early development and helps regulate sleep by acting on specific receptors (MT1 and MT2). Its chronobiotic and hypnotic properties make it effective in treating sleep–wake rhythm disturbances and reducing sleep latency in children, making it a commonly used treatment in pediatric sleep disorders [20]. There are several possible mechanisms by which melatonin may benefit primary headache disorders, including improved circadian rhythm regulation and/or improved sleep [19].

Some observational studies support a role for melatonin in the treatment of migraines in adults. Uncontrolled works in pediatrics also suggest that melatonin may be useful in the treatment of pediatric and adolescent migraine [25]. A multicenter study published in 2020 [26] evaluated whether the use of a combined nutraceutical supplementation based on melatonin, tryptophan, and vitamin B6 may provide a boosted effect in managing nighttime awakenings and in decreasing the frequency of headache episodes. They also made a comparison between this combined product and a nutraceutical containing only melatonin. In relation to headache events, 90% of the children showed some improvement during the two months in which nutritional supplements were used, with no difference between the two nutraceutical products. Concerning the night awakenings, 78.8% of children presented an improvement during the two months of nutritional treatment; in this case, the reduction was statistically significant only in the group of patients who received the combined products.

In an open-label trial in children with primary headaches, melatonin 3 mg twice daily reduced the number, intensity, and duration of headache attacks in 14 out of 21 children [27]. The authors suggested that melatonin might be a safe alternative prophylactic treatment for children with primary headaches, even though these results warrant randomized placebo-controlled trials. Ultimately, there is still no definitive consensus about the therapeutic use of melatonin for headaches in children.

### 3.3. Cluster Headache

Cluster headache (CH) is a primary headache disorder defined by recurrent, unilateral attacks of severe pain, generally occurring at night, typically localized in the orbital or temporal regions, often accompanied by prominent cranial autonomic features [6]. Though well-documented in adults, pediatric CH is rare and frequently underrecognized, especially when it manifests in early childhood or adolescence [28,29]. This rarity, coupled with overlapping symptoms with more common pediatric conditions, contributes to delayed or incorrect diagnosis [30,31]. Consequently, heightened clinical awareness and familiarity with pediatric-specific characteristics are vital for timely identification and treatment.

Due to its scarcity, the epidemiology of pediatric CH is not well characterized. Available studies estimate a prevalence of 0.03% to 0.1% in adolescents [28,32,33]. While CH shows a male predominance in adults, this gender disparity appears less significant in pediatric cases [28,34]. The age of onset spans a wide range, with most patients diagnosed in adolescence, though instances of onset as early as infancy (1 year old) have been recorded, including presentations mimicking sleep disorders [29,35].

A positive family history of headaches appears to be a significant factor in pediatric CH. In particular, a family history of migraines is commonly reported among affected children, potentially indicating a shared genetic susceptibility [33]. Although less frequently noted, a family history of CH itself has also been documented, suggesting that hereditary predisposition may influence both the onset and clinical expression of the disorder in pediatric populations [28,30].

CH pathophysiology is not fully elucidated but is believed to involve trigeminovascular and parasympathetic system activation, with neuropeptides such as calcitonin gene-related peptide (CGRP) and vasoactive intestinal peptide (VIP) contributing [28]. The characteristic circadian rhythm and nocturnal onset suggest hypothalamic involvement, supported by adult neuroimaging studies [34]. Several sleep parameters are affected in CH patients. Data suggests a complex association with REM sleep that may be related to hypothalamic dysregulation [36].

Pediatric CH typically mirrors adult forms in core symptoms, such as severe unilateral periorbital pain and ipsilateral cranial autonomic signs—conjunctival injection, lacrimation, nasal congestion, and ptosis [6,28]. However, age-related differences exist. Autonomic symptoms may be bilateral in children [28,30,37], and attacks often exhibit shorter duration and lower frequency than in adults [30,33,38]. The circadian periodicity, though present, may be less distinct in children [33,38]. Behavioral manifestations during attacks are also distinct: younger children may present with agitation, inconsolable crying, or restlessness, which are frequently misinterpreted as parasomnias or behavioral disorders [29,35]. Unlike adults, pediatric patients often report associated symptoms such as nausea, vomiting, photophobia, and phonophobia with greater frequency [28,33,39]. These overlapping migraine-like features may further complicate diagnosis and delay appropriate treatment. Notably, some pediatric CH cases may exhibit a response to indomethacin, raising diagnostic challenges with paroxysmal hemicrania [29,40]. In some instances, a clear therapeutic response to indomethacin has also supported the diagnostic process in suspected CH cases [29]. Additionally, cases of therapy-resistant CH in childhood have been documented, emphasizing the potential for significant functional impairment and reduced quality of life [39].

Differential diagnosis includes migraine without aura, stabbing headache, other trigeminal autonomic cephalalgias (TACs), trigeminal neuralgia, rhinosinusitis, and secondary causes such as intracranial neoplasms or vascular malformations. Therefore, neuroimaging with vascular studies is advised, despite CH being classified as a primary headache disorder [28,33,34,41,42].

Managing pediatric CH presents challenges due to limited clinical trial data and a lack of pediatric-specific guidelines. Treatment typically adapts adult protocols, with considerations for safety and age-appropriate use. Acute options include high-flow 100% oxygen and triptans, especially subcutaneous sumatriptan and intranasal zolmitriptan, though used with caution due to age-related safety concerns [28,42]. Subcutaneous sumatriptan, although not approved for pediatric use, has shown efficacy in adolescents at doses of 3–6 mg, while zolmitriptan nasal spray demonstrates a favorable safety profile at doses of 5–10 mg. For prevention, verapamil remains the first-line agent, typically administered at 3–10 mg/kg/day in divided doses (up to 600 mg/day), requiring cardiac monitoring in children [28]. Transitional prophylaxis with corticosteroids is frequently employed, such as prednisone at 2 mg/kg/day (up to 100 mg/day) for a short course [28,33,39]. Other prophylactic options include topiramate at 1–2 mg/kg/day, lithium in selected cases, and melatonin (0.1–0.2 mg/kg at bedtime), the latter being particularly relevant due to the circadian component of CH [28,33,39]. Gabapentin and valproate may be considered in refractory or atypical cases, though their use remains off-label and evidence is limited. In some patients, an excellent response to indomethacin therapy has been observed, which may be useful in diagnostic orientation [29].

Non-pharmacological strategies, such as headache diaries, can clarify patterns and identify triggers [40], while multidisciplinary care involving neurologists, pediatricians, and psychologists may optimize diagnosis and adherence in complex or resistant cases [33,38].

The main studies and clinical features about this topic are reported in Table 4.

### 3.4. Hypnic Headache

Hypnic headache (HH) is classified as a primary headache disorder, historically referred to as an “alarm clock headache” or “clockwise headache” [44,45].

Several studies have highlighted the rarity of HH in the pediatric population. One study reported a prevalence of 0.07% based on one case per 1400 headache diagnoses [45]. Another center found HH in 1% of children with strictly unilateral headaches [46]. A more recent population-based study estimated a prevalence of 0.22% for probable HH among 921 children [47].

A recent systematic review [48] focused on pediatric HH identified and described seven pediatric cases across five articles [49,50,51,52,53]. The average age at symptom onset was 10 ± 4.3 years (range: 3–15 years), while the interval between symptom onset and diagnosis averaged 15.8 ± 25.0 months (range: 1–60 months). Most patients (71.4%) experienced nocturnal awakenings due to headache several hours after falling asleep (1–6 h), while in two cases, onset occurred during the early morning (2:00–5:00 a.m.).

Clinical presentation in children differed from adult HH profiles. Pediatric patients often reported pulsating or throbbing pain, contrasting with the dull or pressure-like pain more typical in adults. Additionally, children tended to experience lower attack frequency and shorter durations. In all but one case, attacks lasted more than 10–15 min, with durations up to 60 min, and in a single instance, as long as 5 h. Patients were generally able to return to sleep following headache resolution. The mean frequency of attacks was 12 ± 2.8 days per month (range: 1–25), although one case exhibited a relapsing-remitting pattern, precluding accurate frequency estimation.

Migraine-associated features were mostly absent, though two patients reported occasional nausea (28.6%) and one experienced occasional photophobia (14.3%). Neurological examinations were normal in all subjects. Brain MRI and EEG were conducted in six patients, yielding no pathological findings. Two individuals had a family history of headaches, including one with a maternal history of migraines with aura.

In terms of acute management, only two patients received treatment during attacks: one benefited from acetaminophen within 30–60 min of onset, while the other showed no response to sumatriptan, rizatriptan, or oxygen therapy. Over half of the cohort (57%) did not require pharmacological intervention, as symptoms resolved spontaneously within 30 min.

Prophylactic melatonin was administered in two patients, both of whom exhibited improvement in headache frequency and severity. One patient became symptom-free after six months on 4 mg nightly, and the other achieved remission with a significant reduction in monthly attacks. A further patient, unresponsive to multiple prophylactic agents, achieved complete remission after initiating indomethacin (75 mg at bedtime), which was successfully discontinued after six weeks, with no recurrence over a three-month follow-up. It is worth noting that no overnight polysomnographic studies were conducted in any of the patients reviewed [48].

According to the current ICHD-3 diagnostic criteria [6], HH is defined by recurrent sleep-onset headaches that cause awakening and last up to four hours. These must occur for at least 10 days per month for a minimum of three months, with each episode lasting between 15 min and four hours after awakening, and without associated cranial autonomic symptoms or restlessness. The diagnosis requires exclusion of other ICHD-3 headache entities [6]. The review by Ferretti et al. [48] further indicated that the ICHD-3 criteria were more effective and inclusive in identifying HH in pediatric cases compared to ICHD-2.

The complex interplay between sleep and headaches, particularly HH, remains insufficiently understood, though evidence suggests a bidirectional relationship, in which sleep disturbances can trigger or worsen headache episodes and vice versa [54,55,56]. HH is hypothesized to be a chronobiological disorder [45], with potential involvement of hypothalamic dysfunction, especially the suprachiasmatic nucleus (SCN), and dysregulation of serotonin and melatonin pathways [57,58,59,60,61]. Age-related reductions in SCN cell count and melatonin production may contribute to the increased prevalence of HH in older adults [57,59,61,62]. While initially linked to REM sleep, HH has been found to predominantly occur during NREM sleep, with no definitive sleep stage association, suggesting a possible role of altered sleep microstructure in its pathophysiology [58,63,64,65,66,67].

During diagnostic work-up, alternative primary headaches and secondary causes must be excluded. Guidelines for both acute and preventive management of HH in pediatric patients are limited and largely derived from isolated case studies, small case series, and existing literature analyses. As HH episodes in children typically resolve within 60 min, they frequently remain untreated in the acute phase. Melatonin may be considered a viable prophylactic option in pediatric HH [48].

The considered studies and the main clinical features are summarized in Table 5.

## 4. Discussion

Migraines are a common neurological condition that affects individuals of all ages, including children and adolescents. In the pediatric population, migraines can significantly impact quality of life, daily activities, and overall well-being. Several studies in children suggest an association between migraines and sleep disorders, while lack of sleep is a known trigger factor for migraine attacks [2,4,22].

This review confirms the existence of a strong relationship between migraine and sleep in children. This correlation shows bidirectional aspects: some children with migraines experience migraine attacks during nighttime; on the other hand, sleep disorders are reported as the most frequent comorbidity in children with migraines. Understanding this connection is crucial for developing effective management strategies and improving the health outcomes of young patients suffering from migraines. Studies reported above prove that investigation and treatment of comorbid sleep disorders and sleep hygiene interventions should always be considered in the management of migraine patients, since an improvement in sleep is expected to determine a reduction of headache severity and disability. In some cases, children may not verbalize pain clearly, especially at younger ages, and may instead be irritable, cry inconsolably, or seek parental comfort during the night; for these reasons, some manifestations related to sleep can be missed or misdiagnosed in migraine children. Children with migraine, therefore, need a careful assessment of their sleep habits and of any associated sleep disorders, eventually performing a polysomnography if necessary.

As already mentioned, children affected by migraines with nocturnal attacks and/or sleep disorders are prone to experience more frequent and more severe headaches. This tendency increases their risk of analgesic overuse, emphasizing the importance of prioritizing non-pharmacological treatment strategies in their management.

Improving sleep hygiene plays a key role in reducing both the frequency and duration of migraine episodes in pediatric patients: the literature clearly supports the modification of poor sleep habits as an effective component of migraine prevention. It is therefore crucial to suggest to children and adolescents and to their parents regular sleep schedules, bedtime routines, and reduction of screen time before bed [24].

Furthermore, a cognitive-behavioral therapy focused on sleep and stress management could be a valid therapeutic option in these patients to avoid abuse of analgesics.

A previous review published on headache analyzed the knowledge about the use of melatonin in the treatment of primary headache disorders [19]. There are several possible mechanisms by which melatonin may benefit primary headache disorders, including improved circadian rhythm regulation and/or improved sleep. Some observational studies support a role for melatonin in the treatment of migraines in adults. Uncontrolled works in pediatrics also suggest that melatonin may be useful in the treatment of pediatric and adolescent migraine [25,68]. However, existing studies are limited by small sample sizes, and there is currently no consensus on its therapeutic role in pediatric migraines [20]. Melatonin emerges as a promising therapeutic candidate for pediatric migraine, especially if associated with sleep disorders, demonstrating a favorable safety profile and an absence of significant adverse effects. However, large-scale, randomized controlled trials are warranted to establish its efficacy and inform evidence-based clinical guidelines.

A multicenter study published in 2020 [26,27] evaluated whether the use of a combined nutraceutical supplementation based on melatonin, tryptophan, and vitamin B6 may provide a boosted effect in managing nighttime awakenings and in decreasing the frequency of headache episodes. They also made a comparison between this combined product and a nutraceutical containing only melatonin. In relation to headache events, 90% of the children showed some improvement during the two months in which nutritional supplements were used, with no difference between the two nutraceutical products. Concerning the night awakenings, 78.8% of children presented an improvement during the two months of nutritional treatment; in this case, the reduction was statistically significant only in the group of patients who received the combined products.

For some authors, melatonin might be a safe alternative prophylactic treatment for children with primary headaches, even though these results warrant randomized placebo-controlled trials [27]. Ultimately, there is still no definitive consensus about the therapeutic use of melatonin for headaches in children.

Cluster headache (CH) is a rare form of primary headache. In the pediatric population, only a limited number of cases have been described, making it difficult to identify specific characteristics. Clinically, pediatric CH resembles adult presentations with severe unilateral pain, accompanied by cranial autonomic symptoms, which in children may be bilateral [30,37]. Additionally, migraine symptoms are more commonly reported, contributing to diagnostic challenges [28,33,39]. The clinical presentation can mimic behavioral or sleep disorders. These factors often lead to delayed diagnosis. Neurological examination is usually normal.

Management of pediatric CH is challenging due to the absence of established guidelines. Treatment strategies are generally adapted from adult protocols but adjusted for pediatric safety [28,33,39]. Continued research, especially longitudinal studies and pediatric-focused clinical trials, is essential to enhance our understanding and support the development of targeted guidelines for this disabling disorder in children and adolescents.

The revisions introduced in the ICHD-3 have improved diagnostic sensitivity for HH, yet no new pediatric cases have been reported since 2012, likely due to limited awareness of its clinical presentation in children [52,69]. Developmental age differences may influence symptomatology, suggesting a need for separate diagnostic criteria for pediatric and adult populations [48,70,71]. Current criteria may inadequately capture pediatric HH, which often presents with distinct features such as throbbing pain and lower attack frequency and duration. Despite the rarity and underdiagnosis, melatonin remains the most promising prophylactic treatment in children [48].

At present, knowledge regarding the etiopathogenesis of HH is not entirely certain. A role of the hypothalamus as a regulatory organ of the sleep–wake cycle and involved in pain control has been described in the literature [72,73]. MRI-based assessments have revealed reduced gray matter volume in the posterior hypothalamus of individuals with HH, lending support to the idea of a pathological circadian pacemaker. However, it is still uncertain whether this hypothalamic atrophy precedes HH (as a cause) or results from its occurrence [74,75]. Though HH was initially linked to the REM stage of sleep [64], serial polysomnographic studies have found that only about 20–50% of HH episodes occur during REM, while a larger share—50–70%—take place in non-REM sleep. Moreover, episodes originating in both REM and non-REM phases have been observed in the same patient over a single night, indicating no clear relationship between sleep stage and HH onset [57,66,67,76,77].

Finally, the belief that nighttime headaches warrant neuroimaging is common among clinicians. However, previous studies and this review highlight that primary headaches are a frequent cause of nighttime waking and sleep disruption, and, in most cases, neuroimaging results are normal or reveal incidental abnormalities unlikely to be the cause of the headache [12].

## 5. Conclusions

In the context of secondary headaches, nocturnal awakenings are widely recognized as red flags that may indicate potentially life-threatening underlying conditions. However, the frequency of nocturnal awakenings in children with headaches contrasts with the rarity of emergent intracranial abnormalities in this population, and there is limited consensus on whether such findings can reliably indicate serious secondary pathology [8,12]. Recent studies show that nocturnal awakenings are nonspecific, especially when isolated and found in children with a normal neurological examination [10,11].

The presence of nocturnal awakenings thus appears more likely to be attributable to primary headaches, particularly migraines and, although less frequently, cluster headaches and hypnic headaches. Most primary headaches with nocturnal awakenings could be better controlled by proper sleep hygiene and lifestyle and can be helped by sleep regulatory drugs like melatonin. Some studies show the effectiveness of lifestyle modifications, which can also be associated with drug therapies like monoclonal antibodies, in the treatment of pediatric headaches [78].

It is essential to distinguish the different forms, to perform a thorough medical history that includes pain characteristics, family history, lifestyle habits, sleep hygiene, and a detailed neurological examination in order to determine the most appropriate diagnostic pathway.

It would be valuable to further investigate the current studies on the pathophysiological role of the hypothalamus in the relationship between sleep and nighttime headache, as well as the role of melatonin or other potential pharmacological treatments in their management.

## Figures and Tables

**Table 1:** Diagnostic criteria of migraines, cluster headaches, and hypnic headaches according to ICHD-3 [6].

Migraine	Cluster Headache	Hypnic Headache
A. At least five attacks fulfilling criteria B–D B. Headache attacks lasting 4–72 h (untreated or unsuccessfully treated) C. Headache has at least two of the following four characteristics: 1. Unilateral 2. Location pulsating quality 3. Moderate or severe pain intensity 4. Aggravation by or causing avoidance of routine physical activity (e.g., walking or climbing stairs) D. During headaches, at least one of the following: 1. Nausea and/or vomiting 2. Photophobia and phonophobia E. Not better accounted for by another ICHD-3 diagnosis.	A. At least five attacks fulfilling criteria B–D B. Severe or very severe unilateral orbital, supraorbital, and/or temporal pain lasting 15–180 min (when untreated) C. Either or both of the following: 1. At least one of the following symptoms or signs, ipsilateral to the headache: –Conjunctival injection and/or lacrimation –Nasal congestion and/or rhinorrhea –Eyelid oedema –Forehead and facial sweating –miosis and/or ptosis 2. A sense of restlessness or agitation D. Occurring with a frequency between one every other day and 8 per day E. Not better accounted for by another ICHD-3 diagnosis	A. Recurrent headache attacks fulfilling criteria B–E B. Developing only during sleep, and causing waking C. Occurring on ≥10 days/month for >3 months D. Lasting from 15 min up to 4 h after waking E. No cranial autonomic symptoms or restlessness F. Not better accounted for by another ICHD-3 diagnosis.

**Table 2:** Headaches in the pediatric emergency department: A 5-year retrospective study. Modified from Table 5 of Rossi et al. [10].

Symptom or Clinical Feature	% of Serious Headaches	% of Non-Serious Headaches	*p*-Value
Nocturnal awakenings	0	0.70%	0.18
Visual disturbances	29.10%	1.60%	<0.001
Paraesthesia	0	0.90%	0.63
Cranial nerve palsies	8.30%	0.20%	<0.001
Pupillary abnormalities	12.50%	0.10%	<0.001
Nystagmus	8.30%	0.40%	<0.001
Dysmetria	0	0.20%	0.82
Ataxia	16.70%	0.90%	<0.001
Hyposthenia	12.50%	0.20%	<0.001
Strabismus	8.30%	0.20%	<0.001
Drowsiness	33.30%	0.20%	<0.001
Meningismus	16.70%	0	<0.001

Statistical analysis was undertaken using the Chi-squared test or Fisher’s exact test and multivariate analysis; significance at *p* < 0.05.

**Table 3:** Main studies on migraine and sleep disorders.

Authors, Publication Year	Study Design	Number of Patients Male:Female	Mean Age (Years) Age Range	Main Results
Bruni et al., 1997 [2]	Prospective, case-control	283 (164 M; 119 T), 893 C 144:139	10.11 5–14.3	- Sleep disorders in M and T vs. C: - Bedtime problems: difficulty getting to sleep at night 20.1% M, 17,6% vs. 8.9, *p* < 0.0005; anxiety/fear when falling asleep 30.5% M, 22.7% T vs. 8.2%, *p* < 0.0005; fluid and drugs to facilitate sleep 3% M, 2.5% T vs. 0.7%, *p* < 0.005; - Bad sleep quality 34.8% M, 37.8% T vs. 13.9%, *p* < 0.0005; - nocturnal symptoms: nocturnal hyperkinesia: 45.1% M, 40.3% T vs. 29.0%, *p* < 0.0005; pain of unknown origin during sleep: 4.9% M, 2.5% T vs. 0.6%, *p* < 0.0005; sleep breathing difficulties: 16.5% M, 10.1% T vs. 6.8%, *p* < 0.0005; sleep apnea: 6.1% M, 3.4% T vs. 1%, *p* < 0.0005; - Morning symptoms: restless sleep: 35.4% M, 30.2% T vs. 19.7, *p* < 0.0005; sleep paralysis: 10.9% M, 7.6% T vs. 4.1%, *p* < 0.0005; - Daytime sleepiness: daytime somnolence: 12.2% M, 10.9% T vs. 4.5, *p* < 0.0005; - Recurrent nocturnal attacks 20/283 (7.77%) - Sleep disorders more frequent in nocturnal attack patients vs. diurnal: bedtime struggles 65% vs. 24.7%); night wakings 45% vs. 11.4%; daytime somnolence 60% vs. 10.4%), *p* < 0.0005
Masuko et al., 2014 [5]	Case-control	20 M, 20 C 10:10	9.5 6–12	- 25% migraine patients vs. 0.0% control bruxism.
Armoni Domany et al., 2019 [21]	Retrospective	256 M 104:152	12.65 4.8–18	- 39% obstructive sleep apnea (OSA), 12.9% moderate-severe OSA, 16.8% periodic leg movements, and 2.7% hypoventilation
Bruni et al., 2008 [4]	Retrospective, non-clinical population (students)	1073 subjects 545:528 MG 70 NMG; 135 HFG 868	10.56 8−15	- Timing of the attacks: in the evening, (32.86% MG, 40.00% NMG; in the afternoon (15.71% MG, 18.52% NMG); in the afternoon (15.71% MG, 18.52% NMG); - Sleep problem and sleepiness and circadian preference:-SWPBSMG increased in MG and NMG vs. HFG (*p* = 0.001) - SLS increased in MG vs. NMG and vs. HFG, (*p =* 0.001) - MEQ lower in MG vs. NMG, (*p =* 0.002) and vs. HFG, (*p* = 0.0000)
Voci et al., 2021 [22]	Prospective, questionnaire-based	140 M 54:86	12.1 3–18	- 70% sleep disorders - Vertigo and episodic syndromes 39.2% vs. 18.4%; *p* = 0.021) - >2 attacks/week: 17.5% vs. 10.5% without (10.5%, *p* = 0.031). - Total migraine equivalents: 82.4% versus 60.5% (*p* = 0.007); cyclic vomiting syndrome 18.6% vs. 0.6%; *p* = 0.016; benign paroxysmal vertigo 22.5% vs. 7.9%; *p* = 0.047 - Neuropsychiatric comorbidities: 38.2% vs. 18.4%, *p* = 0.026 - Anxiety or mood disorders: 30.4% vs. 10.5%, *p* = 0.016 - Inefficacy of current acute medications: 5.9% vs. 0.0%, *p* = 0.045
El-Heneedy et al., 2019 [23]	Prospective, case-control	40 M20 C 17:23	11.1 5.4–16.8	- Psychiatric abnormalities (anxious depressed symptoms, withdrawal depressed symptoms, social problems, somatic complaints, and attention problems) more frequent in M vs. C (*p*< 0.05) - Sleep assessment (total sleep time, sleep latency, sleep latency, sleep efficiency) increased more in M vs. C (*p* < 0.05)

When specified, a *p*-value < 0.05 was considered statistically significant. Legend: M: migraine; C: healthy controls; T: tension-type headache; SDs: sleep disorders; MG: migraine group; NMG: non-migraine group; HFG: headache-free group_;_ SWPBSMG: Sleep–Wake Problems Behavior Scale; SLS, Sleepiness Scale; MEQ, Morningness/Eveningness Questionnaire.

**Table 4:** Cluster headaches with sleep awakenings: studies and main clinical features.

Authors	N.	Male:Female	Chronic: Episodic	Onset (Years)	Family History	Location	Attack Duration	Frequency	Migrainous Accompanied Symptoms	Therapy		
										Rescue	Preventive/ Transitional	Indometacin Response
Isik et al., 2002 [29]	3	2:1	Episodic	2–10	Yes (1)	Often not clear	15–60 min	Not specified	Photophobia	/	/	yes
Evers et al., 2002 [42]	1	1:0	Chronic	/	Yes	Unilateral, periorbital	45 min	Every other day	/	O_2_	Refused	/
Majumdar et al., 2009 [38]	11	6:4	Episodic (8)/ Chronic (3)	2–14	6/11	Unilateral, orbital, supraorbital, frontal	72 min	Once a day	Neck stiffness, unilateral photophobia, phonophobia	O2, Dihidroergot., Zolmatriptan	Methysergide, verapamil	Yes (3)
Antonaci et al., 2010 [39]	1	1:0	Episodic	10.5	No	Unilateral, orbital	60–180 min	1–3/day	Phono-photophobia	O_2_	Steroids + cyproheptadine, verapamil	/
Kaciński et al., 2009 [35]	1	/		1	Yes	Sovraorbital	12–240 min	Every 4–5 days- 4/day	Vomiting, nausea, abdominal pain, phono-photophobia	ibuprofen	Verapamil, propanolol, pizotifen	/
Garg et al., 2010 [41]	1	/	Episodic	8	Yes	Forehead and vortex	1–2 h	3–4/day	Vomiting	O_2_	/	yes
Arruda et al., 2011 [30]	3	2:1	Episodic	/	Yes (1)	Periorbital unilateral	40–60 min	Every other day-1–2/day	/	O_2_	Verapamil, prednisone, topiramate, melatonin	yes
Mariani et al., 2014 [43]	11	6:5	Episodic	10	Yes	Orbital (8), fronto-orbital (2), frontal (1)	86 min	1–4/day	Phono-photophobia	/	Prednisone	Yes (1)
Taga et al., 2018 [33]	38	1:1.1	Episodic (31)/ chronic (6)	<13	Yes	Right-sided (17)	15–30 min	3–7/day	Nausea, vomiting, phono-photophobia, osmophobia	/	/	/
Eberhard et al., 2024 [37]	22	1:1.7	Episodic (7)/ Chronic (2)	/	/	/	15–180 min	1/sett- several/day	Nausea, vomiting, phono-photophobia, osmophobia	O_2_, intranasal zolmitriptan, subcutaneous dihydroergotamine, eletriptan, and ubrogepant, subcutaneous sumatriptan	Steroid, propanolol, gabapentin, verapamil, topiramate, biofeedback, occipital nerve block	/

**Table 5:** Characteristics and headache features in reported cases of childhood-onset hypnic headache (modified from Ferretti et al., 2023) [48].

Reference	Gender, Age (y) of Attacks Onset	Time of Attacks	Character of Pain	Localization	Intensity of Pain	Features of Migraine	Duration of Attacks (Min)	Frequency of Attacks (Days/Month)	Family History of Headache	Acute Treatment	Prophylactic Therapy Treatment
Grosberg et al., 2005 [51]	F, 9	5–6 h after falling asleep	Throbbing	Right frontal and temporal	Moderate to severe	None	30	8–12	Negative	No	No
Scagni et al., 2008 [50]	F, 3	2–4 a.m.	Pulsating	Frontal	Severe	None	30–60	1	Positive	Acetaminophen with benefit	No
Cerminara et al., 2011 [49]	M, 7	1–3 h after falling asleep	Na	Fronto-temporal	Moderate	None	20–30	2	Positive (mother: migraine)	No	No
	M, 11	1–2 a.m. and 4–5 a.m.	Dull	Fronto- temporal	Moderate to severe	Occasional nausea	10–20	20–25 (2–3/night)	Negative	No	Melatonin with benefit
	F, 10	1–2 h after falling asleep	Pulsating	Fronto or fronto- temporal	Moderate to severe	Occasional nausea	10–30	10–15 (2–3/night)	Negative	No	Melatonin with benefit
Prakash et al., 2008 [53]	M, 15	1–2 a.m., 3–4 h after falling asleep	Non-throbbing	Left frontal and temporal areas	Moderate to severe	None	30 min, 5 h	not evaluable: relapsing– remitting type disturbance	N/A	Oral sumatriptan, oral rizatriptan, and oxygen inhalation without benefit	Sodium valproate, amitriptyline, duloxetine, naproxen, ibuprofen, and propranolol without benefit. Indomethacin with benefit
Bender, 2012 [52]	F, 15	2.5–3 h after falling asleep	Stabbing	Frontal, temporal, and periocular	N/A	Occasional photophobia	N/A	12–16 (seems to have a seasonal feature)	N/A	na	Treated for OSAS with mandibular advancement oral appliance with benefit

## Data Availability

No new data were created or analyzed in this study. Data sharing is not applicable to this article.

## Abbreviations

The following abbreviations are used in this manuscript:

M	Migraine
MG	Migraine group
NMG	Non-migraine group
HFG	Headache-free group
CH	Cluster headache
HH	Hypnic headache
SCN	Suprachiasmatic nucleus
H	Hours
Y	Years

## References

[1] Onofri A., Pensato U., Rosignoli C., Wells-Gatnik W., Stanyer E., Ornello R., Chen H.Z., De Santis F., Torrente A., Mikulenka P. (2023). Primary Headache Epidemiology in Children and Adolescents: A Systematic Review and Meta-Analysis. J. Headache Pain.

[2] Bruni O., Fabrizi P., Ottaviano S., Cortesi F., Giannotti F., Guidetti V. (1997). Prevalence of Sleep Disorders in Childhood and Adolescence with Headache: A Case-Control Study. Cephalalgia.

[3] Conti R., Marta G., Wijers L., Barbi E., Poropat F. (2023). Red Flags Presented in Children Complaining of Headache in Paediatric Emergency Department. Children.

[4] Bruni O., Russo P.M., Ferri R., Novelli L., Galli F., Guidetti V. (2008). Relationships between Headache and Sleep in a Non-Clinical Population of Children and Adolescents. Sleep Med..

[5] Masuko A.H., Villa T.R., Pradella-Hallinan M., Moszczynski A.J., De Souza Carvalho D., Tufik S., Do Prado G.F., Coelho F.M.S. (2014). Prevalence of Bruxism in Children with Episodic Migraine—A Case-Control Study with Polysomnography. BMC Res. Notes.

[6] Arnold M. (2018). Headache Classification Committee of the International Headache Society (IHS) the International Classification of Headache Disorders, 3rd Edition. Cephalalgia.

[7] Bonfert M., Ebinger F., Blankenburg M., Ertl-Wagner B., Heinen F., Roser T. (2013). Primary versus Secondary Headache in Children: A Frequent Diagnostic Challenge in Clinical Routine. Neuropediatrics.

[8] Tsze D.S., Ochs J.B., Gonzalez A.E., Dayan P.S. (2019). Red Flag Findings in Children with Headaches: Prevalence and Association with Emergency Department Neuroimaging. Cephalalgia.

[9] Raucci U., Parisi P., Ferro V., Margani E., Vanacore N., Raieli V., Bondone C., Calistri L., Suppiej A., Palmieri A. (2023). Children under 6 Years with Acute Headache in Pediatric Emergency Departments. A 2-Year Retrospective Exploratory Multicenter Italian Study. Cephalalgia.

[10] Rossi R., Versace A., Lauria B., Grasso G., Castagno E., Ricceri F., Pagliero R., Urbino A.F. (2018). Headache in the Pediatric Emergency Department: A 5-Year Retrospective Study. Cephalalgia.

[11] Raucci U., Della Vecchia N., Ossella C., Paolino M.C., Villa M.P., Reale A., Parisi P. (2019). Management of Childhood Headache in the Emergency Department. Review of the Literature. Front. Neurol..

[12] Ahmed M.A.S., Ramseyer-Bache E., Taylor K. (2018). Yield of Brain Imaging among Neurologically Normal Children with Headache on Wakening or Headache Waking the Patient from Sleep. Eur. J. Paediatr. Neurol..

[13] Yayıcı Köken Ö., Danış A., Yüksel D., Aksoy A., Öztoprak Ü., Aksoy E. (2021). Pediatric Headache: Are the Red Flags Misleading or Prognostic?. Brain Dev..

[14] De Simone R., Sansone M., Bonavita V. (2020). Headache in Idiopathic Intracranial Hypertension. A CGRP-Dependent Head Pain?. Neurol. Sci..

[15] Gori S., Morelli N., Maestri M., Fabbrini M., Bonanni E., Murri L. (2005). Sleep Quality, Chronotypes and Preferential Timing of Attacks in Migraine without Aura. J. Headache Pain.

[16] Maniyar F.H., Sprenger T., Monteith T., Schankin C., Goadsby P.J. (2014). Brain Activations in the Premonitory Phase of Nitroglycerin-Triggered Migraine Attacks. Brain.

[17] Wu Y.-H., Ursinus J., Zhou J.-N., Scheer F.A., Ai-Min B., Jockers R., van Heerikhuize J., Swaab D.F. (2013). Alterations of Melatonin Receptors MT1 and MT2 in the Hypothalamic Suprachiasmatic Nucleus during Depression. J. Affect. Disord..

[18] Kelman L., Rains J.C. (2005). Headache and Sleep: Examination of Sleep Patterns and Complaints in a Large Clinical Sample of Migraineurs. Headache.

[19] Gelfand A.A., Goadsby P.J. (2016). The Role of Melatonin in the Treatment of Primary Headache Disorders. Headache.

[20] Bruni O., Alonso-Alconada D., Besag F., Biran V., Braam W., Cortese S., Moavero R., Parisi P., Smits M., Van der Heijden K. (2015). Current Role of Melatonin in Pediatric Neurology: Clinical Recommendations. Eur. J. Paediatr. Neurol..

[21] Armoni Domany K., Nahman-Averbuch H., King C.D., Dye T., Xu Y., Hossain M., Hershey A.D., Simakajornboon N. (2019). Clinical Presentation, Diagnosis and Polysomnographic Findings in Children with Migraine Referred to Sleep Clinics. Sleep Med..

[22] Voci A., Bruni O., Ferilli M.A.N., Papetti L., Tarantino S., Ursitti F., Sforza G., Vigevano F., Mazzone L., Valeriani M. (2021). Sleep Disorders in Pediatric Migraine: A Questionnaire-Based Study. J. Clin. Med..

[23] El-Heneedy Y.A.E., Bahnasy W.S., Elahwal S.A., Amer R.A.R., Abohammar S.D.A., Salem H.A.M. (2019). Psychiatric and Sleep Abnormalities in School-Age Children with Migraine. Egypt. J. Neurol. Psychiatry Neurosurg..

[24] Bruni’ O., Galli F., Guidetti V. (1999). Sleep Hygiene and Migraine in Children and Adolescents. Cephalalgia.

[25] Bougea A., Spantideas N., Lyras V.T., Thomaidis T. (2016). Melatonin 4 Mg as Prophylactic Therapy for Primary Headaches: A Pilot Study. Funct. Neurol..

[26] Bravaccio C., Terrone G., Rizzo R., Gulisano M., Tosi M., Curatolo P., Gialloreti L.E. (2020). Use of Nutritional Supplements Based on Melatonin, Tryptophan and Vitamin B6 (Melamil Tripto^®^) in Children with Primary Chronic Headache, with or without Sleep Disorders: A Pilot Study. Minerva Pediatr..

[27] Miano S., Parisi P., Pelliccia A., Luchetti A., Paolino M.C., Villa M.P. (2008). Melatonin to Prevent Migraine or Tension-Type Headache in Children. Neurol. Sci..

[28] Borrelli A., Valeriani M., Monte G., Ursitti F., Proietti Checchi M., Tarantino S., Sforza G., Papetti L. (2024). Cluster Headache: Diagnosis, Management, and Treatment in Pediatric Headache. J. Clin. Med..

[29] Isik U., Neill O., D’cruz F. (2002). Cluster Headaches Simulating Parasomnias. Pediatr. Neurol..

[30] Arruda M.A., Bonamico L., Stella C., Bordini C.A., Bigal M.E. (2011). Cluster Headache in Children and Adolescents: Ten Years of Follow-up in Three Pediatric Cases. Cephalalgia.

[31] Ghosh A., Silva E., Burish M.J. (2021). Pediatric-Onset Trigeminal Autonomic Cephalalgias: A Systematic Review and Meta-Analysis. Cephalalgia.

[32] Gallai B., Mazzotta G., Floridi F., Mattioni A., Baldi A., Alberti A., Sarchielli P., Gallai V., Rasmini P., Besana D. (2003). Cluster Headache in Childhood and Adolescence: One-Year Prevalence in an out-Patient Population. J. Headache Pain.

[33] Taga A., Manzoni G.C., Russo M., Paglia M.V., Torelli P. (2018). Childhood-Onset Cluster Headache: Observations From a Personal Case-Series and Review of the Literature. Headache.

[34] Bastos S.N.M.A.N., Barbosa B.L.F., Silva S.F., Krymchantowski A.G., Jevoux C., Krymchantowski A., Silva-Néto R.P. (2021). Cluster Headache in Children and Adolescents: A Systematic Review of Case Reports. Dev. Med. Child. Neurol..

[35] Kaciński M., Nowak A., Kroczka S., Gergont A. (2009). Cluster Headache in 2-Year-Old Polish Girl. Cephalalgia.

[36] Barloese M.C.J., Jennum P.J., Lund N.T., Jensen R.H. (2015). Sleep in Cluster Headache—Beyond a Temporal Rapid Eye Movement Relationship?. Eur. J. Neurol..

[37] Eberhard S.W., Jackman C.T. (2024). Pediatric Cluster Headache Case Series: Symptomatic Cases and the Migraine Relationship. J. Child. Neurol..

[38] Majumdar A., Ahmed M.A.S., Benton S. (2009). Cluster Headache in Children—Experience from a Specialist Headache Clinic. Eur. J. Paediatr. Neurol..

[39] Antonaci F., Alfei E., Piazza F., De Cillis I., Balottin U. (2010). Therapy-Resistant Cluster Headache in Childhood: Case Report and Literature Review. Cephalalgia.

[40] Klassen B., Dooley J. (2000). Case Report Chronic Paroxysmal Hemicrania-like Headaches in a Child: Response to a Headache Diary. Cephalalgia.

[41] Garg S., Kurup B. (2010). Episodic Cluster Headache: A Rare Diagnosis in Children. Indian Pediatr..

[42] Evers S., Frese A., Majewski A., Albrecht O., Husstedt I. (2002). Age of Onset in Cluster Headache: The Clinical Spectrum (Three Case Reports). Cephalalgia.

[43] Mariani R., Capuano A., Torriero R., Tarantino S., Properzi E., Vigevano F., Valeriani M. (2014). Cluster Headache in Childhood: Case Series from a Pediatric Headache Center. J. Child Neurol..

[44] Newman L., Lipton R., Solomon S. (1990). The Hypnic Headache Syndrome: A Benign Headache Disorder of the Elderly. Neurology.

[45] Dodick D.W., Mosek A.C., Campbell J.K. (1998). The Hypnic (“alarm Clock”) Headache Syndrome. Cephalalgia.

[46] Ramón C., Mauri G., Vega J., Rico M., Para M., Pascual J. (2013). Diagnostic Distribution of 100 Unilateral, Side-Locked Headaches Consulting a Specialized Clinic. Eur. Neurol..

[47] Eliasson J.H., Scher A.I., Buse D.C., Tietjen G., Lipton R.B., Launer L.J., Valdimarsdottir U.A., Gudmundsson L.S. (2020). The Prevalence of Hypnic Headache in Iceland. Cephalalgia.

[48] Ferretti A., Velardi M., Fanfoni C., Di Nardo G., Evangelisti M., Foiadelli T., Orsini A., Del Pozzo M., Terrin G., Raucci U. (2023). Pediatric Hypnic Headache: A Systematic Review. Front. Neurol..

[49] Cerminara C., Compagnone E., Coniglio A., Margiotta M., Curatolo P., Villa M.P., Parisi P. (2011). Hypnic Headache in Children. Cephalalgia.

[50] Scagni P., Pagliero R. (2008). Hypnic Headache in Childhood: A New Case Report. J. Paediatr. Child. Health.

[51] Grosberg B.M., Lipton R.B., Solomon S., Ballaban-Gil K. (2005). Hypnic Headache in Childhood?: A Case Report. Cephalalgia.

[52] Bender S.D. (2012). An Unusual Case of Hypnic Headache Ameliorated Utilizing a Mandibular Advancement Oral Appliance. Sleep Breath.

[53] Prakash S., Dabhi A.S. (2008). Relapsing Remitting Hypnic Headache Responsive to Indomethacin in an Adolescent: A Case Report. J. Headache Pain.

[54] Dosi C., Riccioni A., Della Corte M., Novelli L., Ferri R., Bruni O. (2013). Comorbidities of Sleep Disorders in Childhoodand Adolescence: Focus on Migraine. Nat. Sci. Sleep.

[55] Paolino M.C., Ferretti A., Villa M.P., Parisi P. (2015). Headache and ADHD in Pediatric Age: Possible Physiopathological Links. Curr. Pain Headache Rep..

[56] Parisi P., Verrotti A., Paolino M.C., Ferretti A., Raucci U., Moavero R., Villa M.P., Curatolo P. (2014). Headache and Attention Deficit and Hyperactivity Disorder in Children: Common Condition with Complex Relation and Disabling Consequences. Epilepsy Behav..

[57] Evers S., Goadsby P.J. (2003). Hypnic Headache Clinical Features, Pathophysiology, and Treatment. Neurology.

[58] Obermann M., Holle D. (2010). Hypnic Headache. Expert. Rev. Neurother..

[59] Dodick D.W., Eross E.J., Parish J.M., Silber M. (2003). Clinical, Anatomical, and Physiologic Relationship between Sleep and Headache. Headache.

[60] Rains J.C., Poceta J.S. (2010). Sleep and Headache. Curr. Treat. Options Neurol..

[61] Rains J., Poceta J., Penzien D. (2008). Sleep and Headaches. Curr. Neurol. Neurosci. Rep..

[62] Pascual J. (2009). Other Primary Headaches. Neurol. Clin..

[63] Seidel S., Zeitlhofer J., Wöber C. (2008). First Austrian Case of Hypnic Headache: Serial Polysomnography and Blood Pressure Monitoring in Treatment with Indomethacin. Cephalalgia.

[64] Pinessi L., Rainero I., Cicolin A., Zibetti M., Gentile S., Mutani R., Pinessi L. (2003). Hypnic Headache Syndrome: Association of the Attacks with REM Sleep. Cephalalgia.

[65] Arjona J.M., Jiménez-Jiménez F.J., Vela-Bueno A., Tallón-Barranco A. (2000). Hypnic Headache Associated with Stage 3 Slow Wave Sleep. Headache.

[66] Dolso P., Merlino G., Fratticci L., Canesin R., Valiante G., Coccolo D., Gigli G.L. (2007). Non-REM Hypnic Headache: A Circadian Disorder? A Clinical and Polysomnographic Study. Cephalalgia.

[67] Manni R., Sances G., Terzaghi M., Ghiotto N., Nappi G. (2004). Hypnic Headache PSG Evidence of Both REM-and NREM-Related Attacks. Neurology.

[68] Fallah R., Shoroki F., Ferdosian F. (2015). Safety and Efficacy of Melatonin in Pediatric Migraine Prophylaxis. Curr. Drug Saf..

[69] Liang J.F., Wang S.J. (2014). Hypnic Headache: A Review of Clinical Features, Therapeutic Options and Outcomes. Cephalalgia.

[70] Özge A., Faedda N., Abu-Arafeh I., Gelfand A.A., Goadsby P.J., Cuvellier J.C., Valeriani M., Sergeev A., Barlow K., Uludüz D. (2017). Experts’ Opinion about the Primary Headache Diagnostic Criteria of the ICHD-3rd Edition Beta in Children and Adolescents. J. Headache Pain.

[71] Raieli V., Capizzi M., Marino A., Di Nardo G., Raucci U., Parisi P. (2022). Study on “Atypical” Migraine Auras in the Pediatric Age: The Role of Cortical Spreading Depression and the Physiopathogenetic Hypothesis Arising from Our Clinical Cases. Life.

[72] Lindner D., Scheffler A., Nsaka M., Holle-Lee D. (2023). Dagny Hypnic Headache—What Do We Know in 2022?. Cephalalgia.

[73] Holle D., Obermann M. (2012). Hypnic Headache and Caffeine. Expert. Rev. Neurother..

[74] Holle D., Naegel S., Krebs S., Gaul C., Gizewski E., Diener H., Katsarava Z., Obermann M. (2010). Hypothalamic Gray Matter Volume Loss in Hypnic Headache. Ann. Neurol..

[75] Holle D., Naegel S. (2014). Obermann, Mark Pathophysiology of Hypnic Headache. Cephalalgia.

[76] Holle D., Wessendorf T.E., Zaremba S., Naegel S., Diener H.C., Katsarava Z., Gaul C., Obermann M. (2011). Serial Polysomnography in Hypnic Headache. Cephalalgia.

[77] Liang J.-F., Fuh J.-L., Yu H.-Y., Hsu C.-Y., Wang S.-J. (2008). Clinical Features, Polysomnography and Outcome in Patients with Hypnic Headache. Cephalalgia.

[78] Na J.H., Jeon H., Shim J.E., Lee H., Lee Y.M. (2025). Effectiveness and Safety of CGRP-Targeted Therapies Combined with Lifestyle Modifications for Chronic Migraine in Korean Pediatric Patients: A Retrospective Study. Brain Sci..

